# Scientific Items

**Published:** 1879-08-20

**Authors:** 


### SCIENTIFIC ITEMS.

**How Old Is the World.?**—Geologists, astronomers and physicists have hitherto been baffled in their attempts to set up any satisfactory kind of chronometer which will approximately measure geological time, and thus give us some clue to the antiquity of our globe. It is therefore worth noting that Mr. Mellard Reade, of Liverpool, has lately contributed to the Royal Society a very suggestive paper, in which he endeavors to grapple with the question by employing the limestone rocks of the earth's crust as an index of geological time.

Limestones have been in course of formation from the earliest known geological periods, but it would appear that the later found strata are more calcareous than the earlier, and that there has in fact been a gradually progressive increase of calcareous matter. The very extensive deposition of carbonate of lime over wide areas of the ocean-bottom at the present day is sufficiently attested by the recent soundings of the Challenger.

According to the author's estimate, the sedimentary crust of the earth is at least one mile in average actual thickness, of which probably one-tenth consists of calcareous matter. In seeking the origin of this calcareous matter, it is assumed that the primitive rocks of the original crust were of the nature of gigantic or basaltic rocks. By the disintegration of such rocks, calcareous and other sedimentary deposits have been formed. The amount of lime salts in waters which drain districts made up of granites and basalts is found, by a comparison of analysis, to be on an average about 3.73 parts in 100,000 parts of water. It is further assumed that the excessed areas of igneous rocks, taking an average thoroughout all geological time, will bear to the exposures of sedimentary rocks a ratio of about one to nine. From this and other data Mr. Reade concludes that the elimination of the calcareous matter now found in all the sedimentary strata must have occupied at least 600,000,000 of years. This, therefore, represents the minimum age of the world. The author infers that the formation of the Laurentian, Cambrian and Silurian strata must have occupied about 200,000,000 of years; the old red sand stone, the carboniferous and the poikilitic systems, another 200,000,000 and all other strata the remaining 200,000,000. Mr. Reade is, therefore, led to believe that geological time has been enormously in excess of the limits urged by certain physicists; that it has been ample to allow for all the changes which, on the hypothesis of evolution, have occurred in the organic world.—*London* (Eng.) *Academy.*

**How Deeply Does the Earth Quake?**—The recent earthquake at Virginia City was not noticed at all in the mining depths, but only by the people on the surface. Their famous earthquake of some time ago, which shook down chimneys and fire-walls, cracked brick buildings and did other damage, was merely noticed by some of the miners working in the upper levels, but it did no damage, not even shaking down loose stones and earth. The station-men in the various shafts felt it the strongest, and the deepest point where it was noticed was in the station-tender at 900-foot level of the Imperial-Empire shaft

—900 feet below the surface. He said it felt like a sudden faint throb or pulsation of the air, as though a blast had been let off at a distance above, below, or in some indefinite direction. In some of the mines the shock was not noticed at all, even by station-men. Commenting on this peculiar fact at the time, the Gold Hill News remarked that the earthquake seemed to be an electrical disturbance, proceeding from the atmosphere and not from the depths of the earth.—*Sacramento* (Cal.) *Union.*

**Sphymograph.**—The latest telephonic wrinkle comes from a London physician, who exhibited before the Royal Society the other day what he calls a "sphymograph"—an instrument by which the human pulse, or heart-beat, can be made to register audibly at long distances. In fact, the pulse talks telephonically, and so loudly that when two cells are used the sounds can be heard by an audience of several hundred people. By extending the telephone wires the sounds can also be conveyed long distances, so that a physician in his consulting-room might listen to the heart or pulse of a patient lying in bed (speaking modestly as to distance) a mile or two away.

Dr. Richardson described to the Fellows of the Royal Society that the sounds yielded by the natural pulse resemble the two words "bother it" which, as the Lancet says, is "pretty good for a beginning."

**The Tongue and the Sense of Taste.**—The tasting power of the tongue is not regularly distributed over all parts of that organ. According to the unanimous judgment of physiologists, the back part of the tongue is best qualified for this function, while there is a difference of opinion as to the tip of the tongue. The older observers have repeatedly said that a tasting power in the tip is limited to certain persons, whereas more recent ones affirm its presence in all men. In experimenting on the so-called "reaction-time," Herr Vintschgau lately met with a case of limited tasting power in the tongue tip, and this led him to a thorough investigation of the subject. The observations were made with solutions of chloride of sodium, sugar, quinine and citric acid. The results were as follows: There are persons who are capable of accurately distinguishing all principal tastes with the tip of the tongue alone; others perceive with certainty the qualities of sweetness, saltness, acidity, but less distinctly bitterness. Others, again, can only with great difficulty distinguish tastes with the tip of the tongue; and finally, there are individuals who cannot do this in the least.—*Journal of Chemistry.*

**New Scale.**—By making a miniature of an object, such as a spider line, and examining it with a microscope, Dr. Royston Pigott has found that objects even as small as the millionth of an inch could be seen; and in a late communication to the Philosophical Society, Cambridge, he took exception to the view generally prevailing among opticians, that it is useless to attempt further perfection of the microscope.—*Journal of Chemistry.*

**Nickel.**—The American nickel five-cent piece contains seventy-five per cent. of copper, twenty-five per cent. of nickel, and weighs 77.16 grains.

